# Genome Sequencing of Historical Encephalomyocarditis Viruses from South Africa Links the Historical 1993/4 Savanna Elephant (*Loxodonta africana*) Outbreak to Cryptic *Mastomys* Rodents

**DOI:** 10.3390/pathogens13030261

**Published:** 2024-03-19

**Authors:** Vanessa van Meer, Janusz T. Pawęska, Robert Swanepoel, Antoinette Grobbelaar, Armanda D. Bastos

**Affiliations:** 1 Mammal Research Institute, Department of Zoology and Entomology, University of Pretoria, Hatfield 0028, South Africa; 2 Centre for Emerging Zoonotic and Parasitic Diseases, National Institute for Communicable Diseases, National Health Laboratory Service, Johannesburg 2131, South Africa; 3 Centre for Viral Zoonoses, Department of Medical Virology, University of Pretoria, Pretoria 0001, South Africa; 4 Department of Microbiology and Infectious Diseases, School of Pathology, University of Witwatersrand, Johannesburg 2050, South Africa; 5 Department of Veterinary Tropical Diseases, Faculty of Veterinary Science, University of Pretoria, Onderstepoort 0110, South Africa

**Keywords:** EMCV, *Cardiovirus A*, *Laelaps muricola*, rodents, ectoparasites, phylogenetic analysis, RT-PCR

## Abstract

From 1993 to 1994, 64 free-ranging elephants (*Loxodonta africana*) succumbed to encephalomyocarditis in the Kruger National Park, South Africa, of which 83% were adult bulls. *Mastomys* rodents were implicated as the reservoir host of the *Encephalomyocarditis virus* (EMCV) based on serology and RT-PCR. However, in the absence of sequence-confirmation of both the virus and the rodent host, definitive links between the elephant outbreak strains and rodent reservoir could not be established. In this study, we generate the first reference genome sequences for three historical EMCVs isolated from two *Mastomys* rodents and one *Mastomys*-associated mite, *Laelaps muricola*, in Gauteng Province, South Africa, in 1961. In addition, near-complete genome sequences were generated for two elephant outbreak virus strains, for which data were previously limited to the P1 and 3D genome regions. The consensus sequence of each virus was determined using a PCR-Sanger sequencing approach. Phylogenetic analysis confirmed the three near-identical (99.95–99.97%) *Mastomys*-associated viruses to be sister to the two near-identical (99.85%) elephant outbreak strains, differing from each other at 6.4% of sites across the ~7400-nucleotide region characterised. This study demonstrates a link between *Mastomys*-associated viruses and the historical elephant outbreak strains and implicates *Mastomys* as reservoirs of EMCV in South Africa.

## 1. Introduction

*Encephalomyocarditis virus* (EMCV) (*Picornaviridae*, *Cardiovirus*, *Cardiovirus A*), is an RNA virus comprising three serotypes (EMCV-1, EMCV-2 and EMCV-3; [1,2,3]) that is of economic and wildlife conservation importance. Since the initial isolation of an EMCV-1 virus from a captive gibbon ape in Florida, United States of America (USA), in 1945 [4], subsequent studies confirmed a broad host and geographical range. EMCV has primarily been documented in both wild and domestic pigs [5,6,7,8,9] and murid rodents [10,11,12,13]. Outbreaks in domestic pigs are characterised by sudden myocarditis-associated death, especially in young piglets [6], and reproductive failure in sows, including abortion, stillbirth, and mummification of foetuses [14,15]. The most common sign of EMCV infection in susceptible animals is sudden death [16]. Murid rodents are believed to be reservoirs of the disease [12,16,17] with transmission of the virus occurring indirectly via the faecal–oral route [6,13]. In addition to virus isolation from the serum samples of dogs in China [18] and non-fatal infections in humans [19,20,21], several captive species have succumbed to infection [16,22,23,24,25,26,27]. Only rarely has the virus affected free-ranging wildlife species, the most notable being in African elephants (*Loxodonta africana*), when 64 elephants succumbed to the virus over a nine-month period [11].

Cardioviruses, are RNA viruses with a positive sense single-stranded RNA genome within the family Picornaviridae [1,3]. Picornavirus genomes generally comprise a single open reading frame, approximately 7000–8800 nucleotides (nt) in length, encoding a single polyprotein that is post-translationally cleaved into structural and non-structural proteins [28]. The polyprotein is flanked by the 5′ and 3′ untranslated regions (UTR) and a 3′ poly-A tail. There are four distinct coding regions, viz. the leader (L) protein and three polyprotein (P1, P2 and P3) regions that encode non-overlapping structural and non-structural proteins [1,3,28]. The P1 region encodes four structural viral proteins VP4, VP2, VP3 and VP1, corresponding to the 1A, 1B, 1C and 1D genes [1], whereas the P2 and P3 regions encode three (2A, 2B and 2C) and four (3A, 3B, 3C and 3D) non-structural proteins, respectively. The 3D gene encodes the RNA-dependent RNA polymerase (RdRp) [1,3] and remains the best-represented gene in public databases such as GenBank^®^ (www.ncbi.nlm.nih.gov, accessed on 11 December 2023). This is because, together with the VP3/VP1 gene junction, the 3D gene is frequently targeted in molecular epidemiological studies on encephalomyocarditis, e.g., [2,12,18,20,26,29,30,31,32]. There are presently ~100 partial 3D sequences (>200 nt), ~65 VP3/VP1 junction sequences (>1500 nt), ~65 P1 genome region (>2500 nt) and ~55 full genome (>6800 nt) sequences available for the three EMCV serotypes in the GenBank^®^ database (accessed 11 December 2023).

Historically, South Africa has documented two EMCV outbreaks. The first affecting domestic pigs (*Sus scrofa*) in KwaZulu-Natal (KZN) Province occurred in 1979 [7] and the second in free-ranging African elephants in the Kruger National Park (KNP) occurred from December 1993 to September 1994 [11]. The latter outbreak, which was characterised by adult male-biased mortalities, coincided with a marked increase in myomorph rodent numbers [11]. Virological examination of rodent specimens from the KNP at the time of the outbreak yielded no isolation of EMCV [11]. However, the authors confirmed EMCV antibody presence in 5 of the 12 rodent species tested with an overall prevalence of 25.24%. Seroprevalence was highest in *Mastomys* (95%), which was also the most abundant rodent genus. In addition, RT-PCR tests confirmed EMCV nucleic acid presence in six of the ten antibody-positive *Mastomys* individuals evaluated. Based on this, and on species distribution records available at that time, *Mastomys natalensis* rodents were implicated as the reservoir host of the EMCV outbreak in elephants. However, *Mastomys natalensis* and *Mastomys coucha* occur sympatrically in KNP and are morphologically indistinguishable, forming a cryptic species complex [33,34]. As molecular identification of the EMCV-positive rodents was not performed at the time of the outbreak, and as the virus genome amplicons from *Mastomys* rodents were not sequenced, the link between the elephant outbreak and a specific rodent reservoir remains unknown.

In a prior retrospective study, pig and elephant viruses from the two historical outbreaks of EMCV-1 in South Africa were partially characterised by van Sandwyk et al. [32]. In that study, the authors generated data for the P1 (~3370 nt) and 3D (~245 nt) gene regions of one virus from the 1979 pig outbreak and two viruses from the 1993/4 elephant outbreak. Their results confirmed the presence of two genetically distinct lineages of EMCV in South Africa. The pig outbreak strain clustered with viruses of a wide geographical and host range, whereas the elephant isolates grouped with African Mengoviruses.

In this study, a gap in our understanding regarding the role of indigenous rodents as reservoirs in EMCV transmission and infection in South Africa is addressed by generating the first reference sequences for three historical virus isolates from two *Mastomys* rodents and one *Mastomys*-associated mite, *Laelaps muricola,* sampled in 1961 in Gauteng Province, South Africa. In addition, near-complete genome sequences were generated for two 1994 elephant outbreak viruses, for which only partial genome data were available [32]. Full viral genome sequences were generated using an RT-PCR amplification and Sanger sequencing approach, similar to that employed previously by van Sandwyk and co-workers [32]. Phylogenomic analyses, inclusive of global rodent, pig and elephant EMC viruses were performed to determine the relatedness of historical *Mastomys*-associated viruses and the 1994 elephant outbreak strains from South Africa and their phylogenetic position within the broader EMCV-1 phylogeny.

## 2. Materials and Methods

### 2.1. Study Site and Virus Strains

The three *Mastomys*-associated EMC viruses selected for genetic characterisation were isolated in 1961 from two *Mastomys* individuals that were captured at the Sizwe (formerly known as Rietfontein) Tropical Diseases Hospital grounds, in Edenvale, Gauteng Province, South Africa (26°08′13.3″ S 28°07′28.4″ E), and from one rodent mite, *Laelaps muricola*. Additionally, the two elephant viruses isolated in February 1994 during the outbreak in African elephants in the KNP, for which partial data are available [32] were selected for this study. All isolates are banked at the National Institute for Communicable Diseases (NICD) (26°07′53.2″ S 28°07′03.3″ E) and are designated AN7402/61 and AN7405/61 (*Mastomys* rodent viruses), AR3595/61 (*Mastomys* mite virus), and SPU17/94 and SPU19/94 (two elephant viruses). The viruses were grown on baby hamster kidney (BHK) 21 cells and stored at −70 °C. All virus stocks used in this study were passaged three times on BHK-21 cells.

### 2.2. RNA Extraction and cDNA Synthesis

Viral RNA was extracted from stored supernatant using a commercial kit (QIAamp viral RNA mini kit, Qiagen, Hilden, Germany) under biosafety level (BSL3) containment at the National Institute for Communicable Diseases (NICD), South Africa. Single-stranded viral RNA was reverse transcribed using a random hexanucleotide approach [35], with modification [36]. Briefly, 4.5 µL of the viral RNA template was reverse transcribed in the presence of AMV Reverse Transcriptase reaction buffer (1X, Promega Corporation, Madison, WI, USA), 9µM random hexamers (Promega Corporation, Madison, WI, USA), 0.4 µM dNTPs (Fermentas, Waltham, MA, USA), 10 U Recombinant RNasin^®^ Ribonuclease inhibitor (Promega Corporation, Madison, WI, USA) and 5% DMSO in a final reaction volume of 9 µL. Following an initial denaturation step of 80 °C for 3 min, samples were snap-frozen in liquid nitrogen, transferred to ice, and 10 U of AMV Reverse Transcriptase (Promega Corporation, Madison, WI, USA) and at least 1 U of RNasin were added to each reaction, prior to a one-hour incubation at 42 °C. The enzyme was heat-inactivated at 80 °C for 1 min and the first-strand cDNA products were stored at −20 °C and used as a template for genomic amplification.

### 2.3. Viral Genome Amplification, Purification, and Sequencing

Newly designed primers, as well as those described previously by van Sandwyk et al. [32], were used to amplify overlapping genome fragments varying between ~280 and ~3200 base pairs (bp) in length (Table S1). In addition, the 3D gene region was amplified using primers targeting a 543 bp region as previously described for *Cardiovirus* genome screening in invasive mice (*Mus musculus*) from sub-Antarctic Marion Island [37]. All PCR reactions were prepared in a UV decontaminated laminar flow cabinet, within a dedicated DNA-free laboratory. Genomic amplification reactions were performed in a final reaction volume of 40 µL, containing 1–3 µL of cDNA template, 1X DreamTaqTM Buffer (Fermentas, Waltham, MA, USA), 0.2 µM dNTPs (Fermentas, Waltham, MA, USA), 0.4 µM of each primer and 1.25 U–2.5 U of DreamTaq^TM^ DNA polymerase (Fermentas, Waltham, MA, USA), with the template and *Taq* volume being adjusted according to the size of the target. A negative control containing no cDNA template was included with each primer assay. Touchdown PCRs were performed using an ABI 2720 thermal cycler (Applied Biosystems, Foster City, CA, USA) under assay-specific thermal cycling conditions that were guided by the primer with the lowest melting temperature (Table S1). The annealing temperatures and extension times of each reaction were, respectively, adjusted in a primer pair and amplicon size-specific manner. PCR product size was assessed by 1.5% agarose gel electrophoresis against the GeneRuler^TM^ 1 kb DNA Ladder (ThermoFisher Scientific, Waltham, MA, USA). The gel was stained using Goldview (Geneshun Biotech, Ltd., Guangzhou, China) and visualised under ultraviolet irradiation. Images were captured using a Vilber E-Box gel documentation imaging system (Vilber Lourmat, Collégien, France). PCR amplicons of the expected size were purified directly from the tube or were gel-slice-purified (when one/more non-target bands co-amplified with the intended target) using the Roche High-Pure PCR Product Purification Kit (Roche Diagnostics GmbH, Mannheim, Germany). The BigDye Terminator Cycle Sequencing Ready Reaction Kit (Applied Biosystems, Foster City, CA, USA) was used to perform cycle sequencing with each of the PCR primers at primer-specific annealing temperatures (Table S1). In addition to sequencing with the external forward and reverse PCR primers, internal sequencing primers identified through a primer walking approach (Table S2) were used to generate overlapping sequences for amplicons >1 kbp. Cycle sequencing products were purified using standard ethanol/sodium-acetate precipitation and submitted to the core Sanger sequencing facility of the University of Pretoria, where they were run on an ABI3500xL Genetic analyser (Applied Biosystems, Foster City, CA, USA).

### 2.4. Dataset Compilation and Statistics

Sequence chromatograms were viewed and edited using the Chromas programme embedded in MEGAX v10.2.4 [38]. Individual fragment contigs were generated by the alignment of data generated through bidirectional sequencing, after which terminal regions corresponding to primer-binding sites were removed. Overlapping fragments generated for each virus strain were then aligned to form a contiguous whole genome sequence, inclusive of the 5′-UTR region and flanking leader (L) protein on the 5′ end. The sequences generated for each of the three historical strains were used in NCBI Nucleotide BlastN searches to identify all closely related sequences available on GenBank. Using MEGAX v10.2.4 [38], three datasets were generated for analysis, viz. (i) near-complete genome (5′UTR-L-P1-P2-P3), (ii) VP3/VP1 gene region and (iii) 3D gene region. Sequences were aligned using Muscle embedded in MEGAX v10.2.4 [38] and end-unaligned nucleotides were trimmed resulting in final datasets of the following sizes: (i) 7418 bp, (ii) 1539 bp and (iii) 242 bp. The assembled sequence data for the *Mastomys*-associated viruses, AR3595/61, AN7405/61 and AN7402/61, were each deposited in the NCBI GenBank database under accession numbers OQ858575, OQ858576 and OQ858577, and the viruses isolated from elephants, SPU17/94 and SPU19/94, were submitted under accession numbers OR924201 and OR924200, respectively.

### 2.5. Phylogenetic Analysis

**Figure 1:**
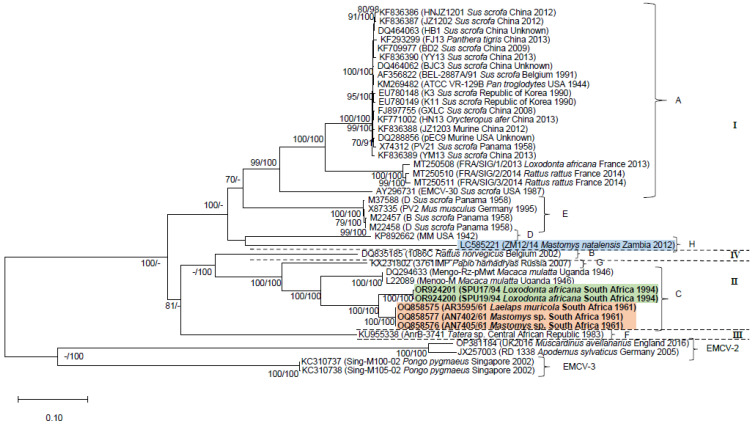
Maximum Likelihood (ML) tree inferred using 7418 nucleotides of the near-complete genome sequences of 41 encephalomyocarditis viruses and the General Time Reversible model of sequence evolution. Nodal support values were estimated from 5000 bootstrap replications from the Maximum Likelihood analysis and from posterior probabilities, expressed as a percentage, from Bayesian Inference (BI). Support values ≥70% and ≥90% are indicated ML/BI next to the relevant nodes. The elephant and *Mastomys*-associated viruses characterised in this study are indicated in bold and highlighted in green and orange, respectively (lineage C). The *Mastomys natalensis* virus from Zambia, characterised by Kishimoto et al. [12] is highlighted in blue (lineage H). Four major evolutionary clades, denoted I–IV, were recovered for EMCV-1 viruses. Representatives of EMCV-2 and EMCV-3 were included for outgroup purposes.

**Figure 2:**
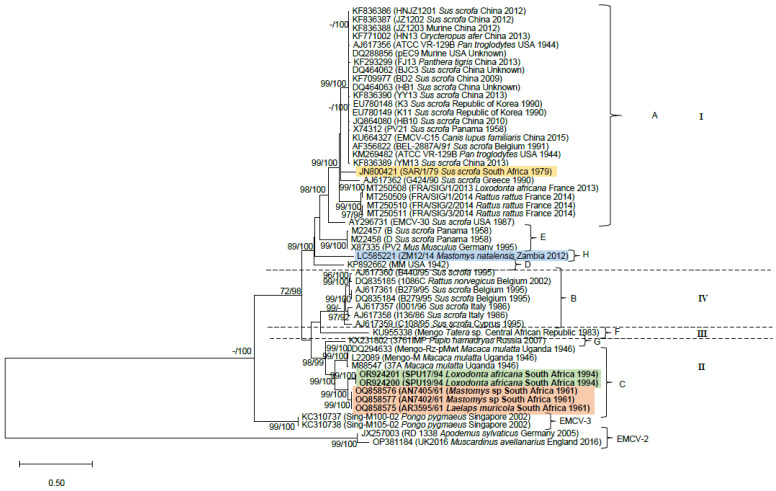
Maximum Likelihood (ML) tree inferred using 1539 nucleotides of the VP3/1 gene region of 52 encephalomyocarditis viruses and the Tamura-Nei model of sequence evolution. Nodal support values were estimated from 5000 bootstrap replications from the Maximum Likelihood analysis and from posterior probabilities, expressed as a percentage, from Bayesian Inference (BI). Support values ≥70% and ≥90% are indicated ML/BI next to the relevant nodes. The elephant and *Mastomys*-associated viruses characterised in this study are indicated in bold and highlighted in green and orange, respectively (lineage C). The *Mastomys natalensis* virus from Zambia, characterised by Kishimoto et al. [12] is highlighted in blue (lineage H), and the 1979 pig outbreak strain from South Africa, characterized by van Sandwyk et al. [32] is highlighted in yellow (lineage A). Four major evolutionary clades, denoted I–IV, were recovered for EMCV-1 viruses. Representatives of EMCV-2 and EMCV-3 were included for outgroup purposes.

**Figure 3:**
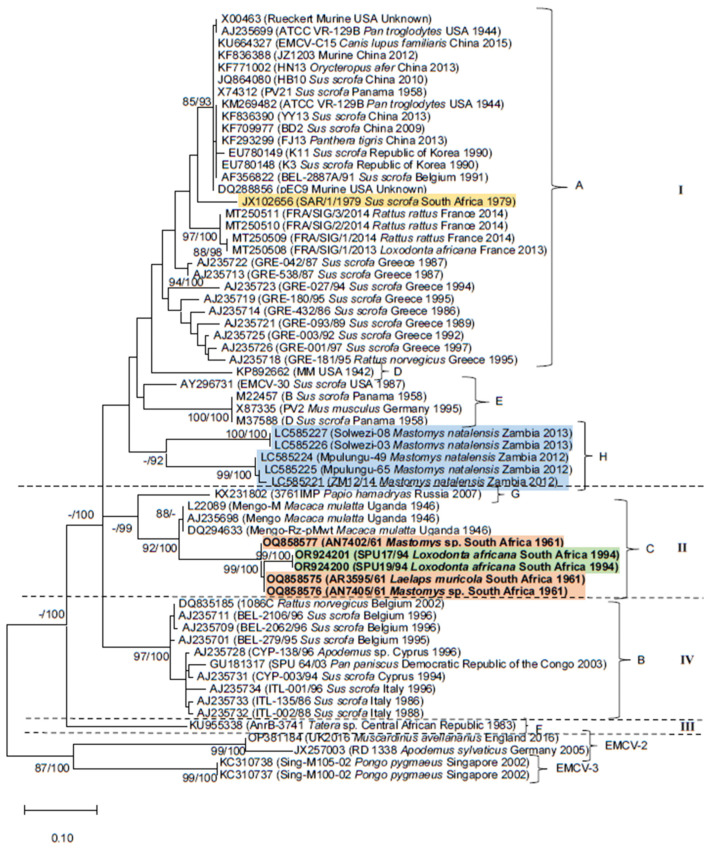
Maximum Likelihood (ML) tree inferred using 242 nucleotides of the 3D gene region of 63 encephalomyocarditis virus sequences and the Kimura-2-Parameter model of sequence evolution. Nodal support values were estimated from 5000 bootstrap replications from the Maximum Likelihood analysis and from posterior probabilities, expressed as a percentage, from Bayesian Inference (BI). Support values ≥70% and ≥90% are indicated ML/BI next to the relevant nodes. The elephant and *Mastomys*-associated viruses characterised in this study are indicated in bold and highlighted in green and orange, respectively (lineage C). The *Mastomys natalensis* virus from Zambia, characterised by Kishimoto et al. [12] is highlighted in blue (lineage H), and the 1979 pig outbreak strain from South Africa, characterized by van Sandwyk et al. [32] is highlighted in yellow (lineage A). Four major evolutionary clades, denoted I–IV, were recovered for EMCV-1 viruses. Representatives of EMCV-2 and EMCV-3 were included for outgroup purposes.

Initial p-distance neighbour-joining trees were inferred for each dataset to identify identical sequences and remove duplicate entries. Phylogenetic analyses, including Maximum Likelihood (ML) and Bayesian Inference (BI), were performed using MEGAX v10.2.4 [38] and MrBayes [39], respectively. Parameters specified for the ML analyses were guided by the best-fit model of sequence evolution identified under the Bayesian Information Criterion (BIC) in MEGAX v10.2.4 [38] for each dataset (Table S3). For BI, the data were partitioned by codon position and four chains, one cold and three heated (default settings), were run for 10 × 10^6^ generations with random starting trees. Trees were sampled every 10,000 generations, after which 25% of the initial run was discarded as burn-in. The convergence of these runs was confirmed using Tracer v1.7.2 [40]. The consensus trees from BI results obtained with, and without, data partitioning by gene region and base position, were viewed in FigTree v1.4.4 [41]. Nodal support values for ML were estimated from 5000 non-parametric bootstrap replications and posterior probabilities ≥0.90 from BI. As topologies were consistent between ML and BI, the posterior probabilities were transferred to the relevant nodes of the ML trees (Figure 1, Figure 2 and Figure 3).

## 3. Results

### 3.1. Genome Characterization

Using Sanger sequencing and read assembly, the near complete genomes (7439 nucleotides (nt)) of the EMC viruses isolated from two *Mastomys* individuals and one *Mastomys*-associated mite were generated. The near-complete genome sequences comprise of a 6882 nt open reading frame (ORF) which encodes a single polyprotein of 2293 amino acids. The ORF is flanked by two untranslated regions (UTR): a 541 nt region corresponding to the 5′-UTR and 16 nt of the 3′-UTR. The three virus strains were near-identical to each other with pairwise nucleotide sequence identity values ranging from 99.95 to 99.97% (Table S4). Mutations occurred at nucleotide positions 3956, 3957, 5074, 6193 and 7300, within the 2B, 2C and 3D gene regions. The predicted amino acids associated with the first and second codon position mutations at sites 3956 and 3957 in 2B are threonine (Thr) for AR3595/61, valine (Val) for AN7402/61 and methionine (Met) for AN7405/61. The nucleotide polymorphisms at sites 5074, 6193 and 7300 correspond to third base position mutations and are silent, encoding leucine (Leu), glutamic acid (Glu) and alanine (Ala), respectively.

The near-complete genomes (7398 nt) of two EMC viruses isolated from cardiac (SPU17/94) and lung (SPU19/94) tissue of two African elephants in 1994 comprised of a 6870 nt open reading frame (ORF), encoding a single polyprotein of 2290 amino acids. The ORF is flanked by a 529 nt region of the 5′-UTR. Based on pairwise nucleotide sequence identity across the genome region (Table S4), the two elephant virus strains were near-identical to each other (99.85%), differing at 11 nucleotide sites. Mutations occurred at nucleotide positions 693, 1101, 1410, 1516, 2871, 4003, 4506, 5280, 5401, 5458 and 5670 within the L, 1B, 1D, 2B, 2C, 3B and 3C gene regions. Four of these mutations occur at the first base position, whilst the remaining seven occur at the third base position. The predicted amino acids associated with the first base position mutations at sites 4003 and 5458 are Alanine (Ala) and Valine (Val), respectively, in SPU17/94, and Threonine (Thr) and Isoleucine (Ile), respectively, in SPU19/94. The nucleotide polymorphisms at the remaining sites are silent mutations. The P1 and 3D regions of the near-complete genome sequences generated in this study were identical to the sequences previously reported by van Sandwyk, et al. [32] for the elephant strains common to both studies.

When considering the publicly available GenBank data as well as the data generated in this study, the two African elephant EMC viruses exhibit the highest nucleotide sequence similarity to the historical *Mastomys*-associated EMCV strains. SPU17/94 exhibits 93.65%, 93.61% and 93.62% nucleotide sequence identity to AR3959/61, AN7402/61 and AN7405/61, respectively, and SPU19/94 exhibits 93.63%, 93.59% and 93.61% nucleotide sequence identity to AR3959/61, AN7402/61 and AN7405/61.

### 3.2. Phylogenetic Analyses

The phylogenies inferred with datasets (i–iii) confirm the sister relationship between the monophyletic lineage comprising historical rodent-associated strains (accession numbers OQ858575, OQ858577 and OQ858576) and the 1994 KNP elephant outbreak strain clade (accession numbers OR924201 and OR924200; Figure 1, Figure 2 and Figure 3). The clade containing the South African elephant and rodent viruses is sister to historical Mengoviruses isolated from *Macaca mulatta* in Uganda in 1946 (88–100% bootstrap support, Figure 1, Figure 2 and Figure 3).

Datasets (i–iii) recovered a total of eight EMCV-1 lineages (Figure 1, Figure 2 and Figure 3) designated A-H on the basis of previous studies [12,13,32,42,43], and four major evolutionary clades denoted I-IV. These lineage and genotype definitions hold across each of the three datasets. Lineage A, spanning Africa, Asia, Europe, and the Americas, although inclusive of a wide range of host species, comprises primarily of pig and rodent viruses. However, strains isolated from a broad range of captive species, viz. the African elephant (*Loxodonta africana*), tiger (*Panthera tigris*), aardvark (*Orycteropus afer*) and chimpanzee (*Pan troglodytes*), and companion animals such as the domestic dog (*Canis lupus familiaris*), that succumbed to EMCV infections [13,18,27,42,44], also cluster within lineage A. Although the EMCV-30 virus (accession number: AY296731) isolated from a pig in the USA [45] groups within lineage A in phylogenies inferred with datasets i and ii (Figure 1 and Figure 2), it clusters within lineage E in the phylogeny inferred using the 3D gene region (dataset iii; Figure 3). Lineage B, which corresponds to evolutionary clade IV, comprises mostly pig and rodent virus strains from Europe and a bonobo (*Pan paniscus*) from the Democratic Republic of the Congo [26]. Lineage C comprises EMCV strains isolated from various indigenous hosts on the African continent, including *Mastomys* rodents and African elephants from South Africa, that were characterised in this study, and rhesus monkeys (*Macaca mulatta*) from Uganda [42,46]. Lineage D comprises only one representative taxon, the EMC virus strain MM isolated in 1942 in the USA. Lineage E comprises EMCV strains isolated from pigs in Panama and *Mus musculus* in Germany [47,48,49]. Lineage F comprises a strain of Mengovirus isolated from *Tatera* (now *Gerbilliscus*) from the Central African Republic in the early 1980s [50] and constitutes the sole representative of evolutionary clade III. An EMCV strain isolated from a hamadryas baboon (*Papio hamadryas*) in Russia forms lineage G [43]. Lastly, lineage H comprises strains of EMCV isolated from *Mastomys natalensis* in Zambia, in which host species identification was confirmed by molecular typing [12].

All phylogenetic trees demonstrate links between rodent-associated EMC viruses and viruses from non-rodent hosts in various parts of the world, including South Africa (lineage C). In lineage A, murid rodent viruses from the USA cluster with EMC viruses isolated from a wide range of hosts, globally (Figure 1, Figure 2 and Figure 3). Similarly, viruses from *Rattus rattus* are sister to the virus isolated from an African elephant from France (Figure 1, Figure 2 and Figure 3) and are identical to one another across the VP3/VP1 and 3D gene regions. 3D gene sequencing of a *Rattus norvegicus* virus from Greece links with viruses from outbreaks in pigs in Greece (Figure 1). In lineage B, a virus from *Rattus norvergicus* clusters with pig viruses from Italy and Belgium (Figure 2 and Figure 3) and a virus isolated from *Apodemus* clusters with pig viruses from Italy (Figure 3). Lineage E, comprising a *Mus musculus* virus from Germany is sister to pig viruses from Panama (Figure 1, Figure 2 and Figure 3); these viruses are identical to one another across the partial 3D gene region analysed.

## 4. Discussion

Eight EMCV-1 lineages (A–H) have been described to date [12,13,18,27,43]. Genetic characterisation of the three rodent-associated EMC viruses revealed the three viruses to be near-identical and the two elephant viruses to be near-identical to each other. The virus, AN7405/61, isolated from *Mastomys*, exhibits greater sequence identity to the virus isolated from *Laelaps muricola* (AR3595/61) than to the other isolate from a *Mastomys* rodent (AN7402/61) that was captured within the same five-day period. The historical *Mastomys* and elephant-associated viruses fall within lineage C, a lineage consistently recovered in all three datasets (i–iii) analysed. In contrast, Kishimoto et al. [12] found that the EMCV isolated from *M. natalensis* in Zambia clustered within a distinct EMCV-1 lineage (H), suggesting that the *Mastomys*-associated EMCV in Zambia is distinct from the strains isolated from *Mastomys* rodents in Gauteng Province, South Africa. The elephant and *Mastomys*-associated viruses from South Africa form a monophyletic lineage that is sister to Mengoviruses isolated from rhesus macaques in Uganda, supporting previous suggestions of a geographically distinct African lineage [32], as all lineage C viruses are of African origin. This lineage comprises EMCV strains isolated from three indigenous hosts, including *Mastomys* rodents and African elephants (this study; [32]) from South Africa, as well as rhesus monkeys (*Macaca mulatta*) from Uganda [42,46]. Lineages A-H are, for the most part, consistently recovered with all three datasets (i–iii), although the phylogenetic placement of the Central African Republic AnrB-3741 strain from *Tatera* (accession number: KU955338), which solely constitutes lineage F is variable. This virus constitutes a well-supported evolutionary clade (III) in the partial 3D phylogeny (Figure 3) that is basal to the monophyletic lineage comprising of clades I, II and IV, which constitute a soft polytomy. The phylogenetic placement of the EMCV-30 strain originating from *Sus scrofa* in the USA is also inconsistent across phylogenies. This virus clusters with lineage A viruses in phylogenies (i and ii) and within lineage E in the 3D phylogeny (Figure 3, dataset iii). This suggests the likely presence of a recombinational hotspot between the 2A and 3D regions, which is consistent with the results obtained for other picornaviruses [28,51,52]. High mutation rates have been recorded in picornaviruses, with substitution rates ranging from 1.61 × 10^−3^ to 5.73 × 10^−3^ for EMCV and human enterovirus B (CVB4), respectively, across the VP1 gene [53]. These high mutation rates result in the formation of quasispecies, in which the viral population resembles a cloud containing a substantial variety of genomes that is centred around the original consensus genome [54] which can be recovered by PCR amplification and nucleotide sequencing of all variants present in the cloud [55].

The link between elephant viruses and rodent-associated EMC viruses from South Africa is clear based on the sister relationships (99–100% bootstrap support) and the high nucleotide sequence identity recovered in this study. Similarly, a link between viruses shed by rats trapped in/near elephant enclosures in France was demonstrated through genome sequencing of viruses from captive elephants that succumbed to EMCV infection in captivity [13]. Similar links between rodent viruses and those isolated from other species occur in lineages A, B and E, including viruses from *Rattus norvegicus* clustering with pig viruses from both Belgium and Italy; an *Apodemus* sp. virus from Cyprus linked to pig viruses in Cyprus and Italy; a *Rattus norvegicus* virus and pig viruses from Greece cluster together; a *Mus musculus* virus from Germany clusters with pig strains from Panama; and murine viruses isolated in the USA and China cluster with various other global strains. While most examples show links between rodents and susceptible species occurring within the same geographical area, the links between rodents and susceptible species in other parts of the world are not surprising given that the geographic ranges of invasive species, such as *Mus* and *Rattus*, are increasingly driven by human-mediated movement [56,57]. Overall, the phylogenies demonstrate links between rodent-associated EMC viruses and a broad range of susceptible species, suggesting that virus spillover from murid rodents is a worldwide occurrence and that multiple rodent genera are involved.

Phylogenetic analysis of the first generated rodent-associated EMCV strains in South Africa has demonstrated that the viruses isolated from *Mastomys* and a *Mastomys*-associated *Laelaps* mite are phylogenetically more closely related to historical outbreak strains from African elephants in South Africa than global rodent-associated EMCV strains. The sister relationship and high nucleotide sequence identity of these lineage C viruses suggest an endemic circulation of the virus in *Mastomys* rodents from South Africa. This is supported by the detection of EMCV-neutralizing antibodies and viral nucleic acids in *Mastomys* from the KNP [11]. Whilst the genetic relatedness of South African elephant virus- and *Mastomys*-associated virus strains supports this hypothesis, the identity of the *Mastomys* rodent host species remains unclear. Renewed rodent sampling efforts to simultaneously detect the virus and type of the host species are needed. Differentiation of morphologically indistinguishable cryptic species is readily achieved using a genetic approach, such as cytochrome *b* gene sequencing, and is particularly important in South Africa, where both rodent diversity and the presence of cryptic species complexes are high [58]. The value of this combined approach was demonstrated in a recent study in Zambia which implicated *M. natalensis* as a possible reservoir of EMC lineage H viruses [12].

EMCV was isolated from a *Laelaps muricola* mite sampled from a *Mastomys* rodent in 1961. *Laelaps muricola* is a generalist rodent mite that commonly occurs on *M. coucha* and *M. natalensis* [59] and hosts pathogens such as *Rickettsia* [60]. Isolation of EMCV from this ectoparasite species is consistent with historical reports of virus isolation from several arthropods, including mosquitoes [61,62,63], ticks (*Ixodes petauristae* and *Haemaphysalis spinigera*) [64] and even parasitic crustaceans (*Porocephalus armillatus*) [17]. Subsequent experimental transmission attempts using mosquitoes (*Aedes aegypti*) have failed [62,65], suggesting that prior detection of the virus in mosquitoes was likely due to virus acquisition through feeding on an infected host. Accordingly, the potential for the vector-borne transmission of EMCV has received limited attention since the 1950s and 1960s studies investigating this route of transmission.

EMCV outbreaks are not uncommon in domestic pigs [6,7,8,9], and sporadic outbreaks continue to occur in captive animals [2,16,23,25,66,67]. Only rarely have outbreaks been recorded in free-ranging, semi-wild or wild animals [11,26,27]. These outbreaks have been recorded globally and often coincide with marked increases in myomorph rodent populations [8,11,13,22,23,25]. EMCV infection can have severe economic implications as outbreaks result in mass mortalities of pigs on farms [9]. Additionally, implications for conservation occur when captive/semi-captive IUCN red-list (https://www.iucnredlist.org, accessed on 11 December 2023) species such as the South China tiger (*Panthera tigris amoyensis*) [27] in ex situ breeding facilities succumb to the virus. The implications of mass mortality events triggered by pathogens, including EMCV infection [11] for small populations of savanna elephants (*Loxondonta africana*) which are unable to withstand the loss of many individuals has been highlighted [68]. It is therefore important to control the spread of EMCV. This can be achieved through available emergency vaccination in captive and wild populations [69,70,71] and effective rodent–pest control programs that limit transmission of EMCV by rodents via the faecal–oral route by removing them from captive environments and pig operations [13,72].

In Africa, multimammate mice of the genus *Mastomys* have been implicated as the reservoir hosts of EMCV [11,12]. By generating the first reference sequences for EMC viruses isolated from *Mastomys* rodents within South Africa, as well as the first complete sequences for the 1994 KNP elephant EMC viruses, we demonstrated the presence of an EMC lineage C virus in indigenous *Mastomys* rodents three decades prior to the male-biased elephant mortality event in elephants, which was caused by a closely related strain. These results confirm the role of *Mastomys* in EMCV transmission and infection in South Africa. However, it is still unclear whether *M. natalensis* or *M. coucha* is the most likely reservoir host of EMCV as rodent typing was not conducted by Grobler et al. [11] in the KNP, nor were the *Mastomys* individuals associated with the three EMCV strains characterised in this study typed. Thus, in order to understand the maintenance of EMCV, disease epidemiology and the potential for spillover to domestic and wild animals from indigenous rodents in South Africa, rodent reservoir hosts of EMCV must be identified. This can be achieved through expanded studies involving sampling *M. coucha* or *M. natalensis* for EMCV presence in combination with the virus and rodent host typing.

This study reported the genetic characterisation of the first reference sequences of *Mastomys*-associated EMC viruses from South Africa as well as the complete elephant EMCV sequences, and has provided valuable reference genome data, confirming the role that indigenous rodents play in viral transmission and infection. The presence of an African clade and confirmation of closely related EMC viruses of *Mastomys* and elephant origin further highlight the likelihood of virus spillover from these rodents to elephants, and represents an important step in closing a notable gap in our understanding of EMCV epidemiology in South Africa.

## Data Availability

Publicly available datasets were analysed in this study and can be found here: [https://www.ncbi.nlm.nih.gov/genbank/] (accessed on 11 December 2023).

## Supplementary Materials

The following supporting information can be downloaded at https://www.mdpi.com/article/10.3390/pathogens13030261/s1. Table S1: Primer pairs used to amplify and sequence overlapping coding and non-coding genome regions for five historical encephalomyocarditis viruses from South Africa, inclusive of two elephants (SPU17/94 and SPU19/94), one *Laelaps* mite (AR3959/61) and two *Mastomys* (AN7402/61 and AN7405/61) strains. Table S2: Internal primers, designed during the course of this study, to generate overlapping genome region sequences for both strands of each amplicon for three historical rodent-associated EMC viruses in South Africa; Table S3. Summary statistics for the three EMCV-1 datasets (i–iii) used for phylogenetic inference in this study; Table S4. Matrix showing the pairwise nucleotide sequence identity (%) between five historical encephalomyocarditis viruses from South Africa inclusive of two elephants (SPU17/94 and SPU19/94), one *Laelaps* mite (AR3959/61) and two *Mastomys* (AN7402/61 and AN7405/61) strains.
