# Supplementary material for: Genome Sequencing of Historical Encephalomyocarditis Viruses from South Africa Links the Historical 1993/4 Savanna Elephant (Loxodonta africana) Outbreak to Cryptic Mastomys Rodents
Source: Pathogens. 2024 Mar 19;13(3):261. doi: 10.3390/pathogens13030261 (PMC10974613; doi:10.3390/pathogens13030261)
Supplement: Supplementary file 1 [file pathogens-13-00261-s001.zip › pathogens-2828204-supplementary.pdf]

Table S1. Primer pairs used to amplify and sequence overlapping coding and non-coding genome regions for five historical encephalomyocarditis viruses from South Africa inclusive of two elephants (SPU17/94 and SPU19/94), one *Laelaps* mite (AR3959/61) and two *Mastomys* (AN7402/61 and AN7405/61) strains.

Primer set and orientation (F: Forward/R: Reverse)	Reference	Primer binding site*	Historical virus strains amplified	Expected amplicon size	Targeted genome region	Touch down PCR annealing temperatures and the number of cycles
EMC-AB1: GGCCGAAGCCGCTTGGAATA (F)<br>EMC-AB2: ACGTGGCTTTTGGCCGCAGA (R)	van Sandwyk *et al*. (2013)<br>van Sandwyk *et al*. (2013)	219-238<br>484-503	SPU17/94;<br>SPU19/94	285	5'UTR	59°C (2x), 57°C (3x), 55°C (35x)
EMC-AB1: GGCCGAAGCCGCTTGGAATA (F)<br>1356R-AB: TGGGTGTTTGTGACCGTGTT (R)	van Sandwyk *et al*. (2013)<br>van Sandwyk *et al*. (2013)	219-238<br>1232-1251	All	1037	5'UTR-1B	61°C (2x), 59°C (3x), 57°C (35x)
687F-AB: GCTCTCCTCAAGCGTATTCA (F)<br>1356R-AB: TGGGTGTTTGTGACCGTGTT (R)	van Sandwyk *et al*. (2013)<br>van Sandwyk *et al*. (2013)	581-600<br>1232-1251	SPU17/94;<br>SPU19/94	1886	5'UTR-1B	50°C (2x), 48°C (3x), 46°C (35x)
687F-AB: GCTCTCCTCAAGCGTATTCA (F)<br>2113R-AB: CAGTTCCCACCATGCGGAAGTG (R)	van Sandwyk *et al*. (2013)<br>van Sandwyk *et al*. (2013)	581-600<br>2444-2465	All	1886	5'UTR-1C	57°C (2x), 55°C (3x), 53°C (35x)
VP4-F1: CGCCGATCAAGATACGGAGGA (F)<br>2B-RM: CGGCAGTAGGGTTTGAGCCATT (R)	This study<br>van Sandwyk *et al*. (2013)	926-946<br>4070-4091	AN7402/61	3166	L-2B	60°C (2x), 58°C (3x), 56°C (35x)
1307F-AB: TCAGACCGAGTGTCTCAAGA (F)<br>2113R-AB: CAGTTCCCACCATGCGGAAGTG (R)	van Sandwyk *et al*. (2013)<br>van Sandwyk *et al*. (2013)	1202-1221<br>2444-2465	All	1264	1B-1C	59°C (2x), 57°C (3x), 55°C (35x)
1307F-AB: TCAGACCGAGTGTCTCAAGA (F)<br>1720R-AB: CTTGGACCATCTGTTGTCCAT (R)	van Sandwyk *et al*. (2013)<br>van Sandwyk *et al*. (2013)	1317-1336<br>1710-1730	AR3595/61;<br>AN7402/61;<br>AN7405/61	414	1B	55°C (2x), 53°C (3x), 51°C (35x)
1904F-AB: GCTTCCTGGACTTTGGTGAT (F)<br>2B-RM: CGGCAGTAGGGTTTGAGCCATT (R)	van Sandwyk *et al*. (2013)<br>van Sandwyk *et al*. (2013)	1914-1933<br>4070-4091	AR3595/61;<br>AN7402/61;<br>AN7405/61	2178	1B-2B	56°C (2x), 54°C (3x), 52°C (35x)
2246MF-JvS: GTACCGTGGATCACTAGTCTA (F)<br>3204MR-JvS: GACAGCAGGTAGGACAGACAA (R)	van Sandwyk *et al*. (2013)<br>van Sandwyk *et al*. (2013)	2248-2268<br>3212-3232	SPU17/94;<br>SPU19/94	985	1C-1D	58°C (2x), 57°C (3x), 56°C (35x)
2246MF-JvS: GTACCGTGGATCACTAGTCTA (F)<br>2B-RM: CGGCAGTAGGGTTTGAGCCATT (R)	van Sandwyk *et al*. (2013)<br>van Sandwyk *et al*. (2013)	2248-2268<br>3954-3976	SPU19/94	1729	1C-2B	56°C (2x), 54°C (3x), 52°C (35x)
VP3-FM: CACTTCCGCATGGTGGGAACTG (F)<br>2B-RM: CGGCAGTAGGGTTTGAGCCATT (R)	van Sandwyk *et al*. (2013)<br>van Sandwyk *et al*. (2013)	2444-2465<br>3954-3976	SPU17/94;<br>SPU19/94;<br>AR3595/61;<br>AN7402/61	1533	1C-2B	57°C (2x), 55°C (3x), 53°C (35x)

VP3-FM: CACTTCCGCATGGTGGGAACTG (F)<br>EMC2C-IR: CGTCCTCGTTGGCATCGAGCAA (R)	van Sandwyk *et al*. (2013)<br>This study	2444-2465<br>4019-4040	SPU17/94	1597	1C-2A	64°C (2x), 62°C (3x), 60°C (35x)
EMC2B-F4: CCACAAGGGATTGGAGGTTAGA (F)<br>EMC2C-IR: CGTCCTCGTTGGCATCGAGCAA (R)	This study<br>This study	3526-3547<br>4019-4040	SPU19/94	515	2A-2B	60°C (2x), 58°C (3x), 56°C (35x)
EMC-2BF2: GGTCAGTTGTGGGCTGAAACA (F)<br>EMC2C-IR: CGTCCTCGTTGGCATCGAGCAA (R)	This study<br>This study	3744-3764<br>4019-4040	SPU19/94	297	2A-2B	61°C (2x), 59°C (3x), 57°C (35x)
EMC-2BF2: GGTCAGTTGTGGGCTGAAACA (F)<br>EMC3C-IR: GTGTGACTAACACCACGCACTAC (R)	This study<br>This study	3744-3764<br>5810-5832	SPU17/94	2089	2A-3AB	60°C (2x), 58°C (3x), 56°C (35x)
EMC-2BF3: GCAGGATATTTCACGGATCTC (F)<br>EMC-3DR3: CCCAATCTCTCTGCTGCATAT (R)	This study<br>This study	4026-4046<br>6848-6868	AR3595/61; AN7402/61; AN7405/61	2843	2B-3D	60°C (2x), 58°C (3x), 56°C (35x)
4049-JvSF: GGAGCGGCAGTGTCATCAAT (F)<br>EMC3C-IR: GTGTGACTAACACCACGCACTAC (R)	This study<br>This study	4053-4072<br>5810-5832	SPU17/94; SPU19/94	1780	2B-3AB	59°C (2x), 57°C (3x), 55°C (35x)
4518-FAB: CGGAATGGAATGGCTGCCTATGT (F)<br>EMC3C-IR: GTGTGACTAACACCACGCACTAC (R)	This study<br>This study	4641-4663<br>5810-5832	SPU17/94; SPU19/94	1192	2C-3C	62°C (2x), 60°C (3x), 58°C (35x)
EMC2C-IF: GCGCCAGTCAGTATATTCTCTTC (F)<br>EMC3C-IR: GTGTGACTAACACCACGCACTAC (R)	This study<br>This study	4770-4792<br>5810-5832	SPU17/94; SPU19/94	1063	2B-3AB	61°C (2x), 59°C (3x), 57°C (35x)
EMC-3CF1-VvM: GCCCCTATTGATTTTGTGTAT (F)<br>EMC-3D2R: CCGGTTGGGTCTGGAACTT (R)	This study<br>This study	5696-5716<br>6963-6981	SPU17/94; SPU19/94	1286	3C-3D	54°C (2x), 52°C (3x), 50°C (35x)
EMC-3CF2-VvM: GCCGCGTCAATAATTTCACAA (F)<br>EMC-3D2R: CCGGTTGGGTCTGGAACTT (R)	This study<br>This study	6202-6222<br>6963-6981	SPU17/94; SPU19/94	780	3C-3D	58°C (2x), 56°C (3x), 54°C (35x)
6453F-VVM: CCCTCCAGTGTTTAGAATGGTT (F)<br>EMC-3DRAB: CTTACCGGGTAACGCGTTGT (R)	This study<br>van Sandwyk *et al*. (2013)	6451-6472<br>7774-7793	SPU19/94	1343	3D	58°C (2x), 56°C (3x), 54°C (35x)
6793F-VvM: CCCACCATTTGAGCATTGCAT (F)<br>7256R-VvM: CGCCATAAGACAAGATCTTCACAT (R)	This study<br>This study	6793-6813<br>7233-7256	SPU17/94; SPU19/94	464	3D	58°C (2x), 56°C (3x), 54°C (35x)

*Primer binding sites correspond to reference sequence L22089 (Mengo isolate M *Macaca mulatta* Uganda 1946).

Table S2: Internal primers, designed during the course of this study, to generate overlapping genome region sequences for both strands of each amplicon for three historical rodent-associated EMC viruses in South Africa.

**Primer name and Orientation (F: Forward/R: Reverse)**	**Primer binding site***	**Amplified virus strains**
1D-R2: 5' - GGCAAATCCTGACTCCAAACTC - 3' (R)	2590-2611	AN7402/61
EMC-AB2: 5' - ACGTGGCTTTTGGCCGCAGA - 3' (R)	598-617	AR3595/61; AN7402/61; AN7405/61
EMC2C-IR: 5' - CGTCCTCGTTGGCATCGAGCAA - 3' (R)	4019-4040	AR3595/61; AN7402/61; AN7405/61
EMC3C-IR: 5' - GTGTGACTAACACCACGCACTAC - 3' (R)	5810-5832	AR3595/61; AN7402/61; AN7405/61
EMC2C-IF: 5' - GCGCCAGTCAGTATATTCTCTTC - 3' (F)	4770-4792	AR3595/61; AN7402/61; AN7405/61

*Primer binding site determined using the reference sequence L22089 (Mengo isolate M *Macaca mulatta* Uganda 1946).

Table S3. Summary statistics for the three EMCV-1 datasets (i-iii) used for phylogenetic inference in this study.

					**Average nucleotide composition (%): empirical/uncorrected**					
**Dataset**	**No. taxa**	**Length (bases)**	**Va sites**	**Pi sites**	**T**	**C**	**A**	**G**	**R**	**Best-fit model of sequence evolution (under BIC)**
(i) Near-complete genome	41	7418	3511	3189	25.7	24.3	26.4	23.6	1.57	GTR+G+I (G=0.67, I=0.39)
(ii) VP3/1 gene	52	1539	778	729	27.1	27.1	24.2	21.6	1.42	TN93+G+I (G=0.91, I=0.44)
(iii) 3D gene	63	242	105	94	27.2	24.7	27.7	20.4	2.3	K2P+G (G=0.24)

BIC: Bayesian information criterion; No.: Number; V: variable; Pi: parsimony-informative; R: transition: transversion ratio (R). GTR: General Time Reversible; TN93: Tamura-Nei; K2P: Kimura 2-parameter; G: Gamma distribution; I: proportion of invariant sites.

Table S4. Matrix showing the pairwise nucleotide sequence identity (%) between five historical encephalomyocarditis viruses from South Africa inclusive of two elephant (SPU17/94 and SPU19/94), one *Laelaps* mite (AR3959/61) and two *Mastomys* (AN7402/61 and AN7405/61) strains.

	**SPU19/94**	**SPU17/94**	**AR3595/61**	**AN7402/61**	**AN7405/61**
**SPU19/94**					
**SPU17/94**	99.8513				
**AR3595/61**	93.6334	93.6469			
**AN7402/61**	93.5929	93.6064	99.9459		
**AN7405/61**	93.6064	93.6199	99.9730	99.9459	
